# Context-dependent hyperactivity in *syngap1a* and *syngap1b* zebrafish models of SYNGAP1-related disorder

**DOI:** 10.3389/fnmol.2024.1401746

**Published:** 2024-07-10

**Authors:** Sureni H. Sumathipala, Suha Khan, Robert A. Kozol, Yoichi Araki, Sheyum Syed, Richard L. Huganir, Julia E. Dallman

**Affiliations:** ^1^Department of Biology, University of Miami, Coral Gables, FL, United States; ^2^Department of Biological Sciences, North Carolina State University, Raleigh, NC, United States; ^3^Department of Biological Sciences, St. John’s University, Queens, NY, United States; ^4^Department of Neuroscience and Kavli Neuroscience Discovery Institute, Johns Hopkins University School of Medicine, Baltimore, MD, United States; ^5^Department of Physics, University of Miami, Coral Gables, FL, United States

**Keywords:** zebrafish, sensory-processing, autism, syngap1, haploinsufficiency mutant profiling

## Abstract

**Background and aims:**

SYNGAP1-related disorder (SYNGAP1-RD) is a prevalent genetic form of Autism Spectrum Disorder and Intellectual Disability (ASD/ID) and is caused by *de novo* or inherited mutations in one copy of the *SYNGAP1* gene. In addition to ASD/ID, SYNGAP1 disorder is associated with comorbid symptoms including treatment-resistant-epilepsy, sleep disturbances, and gastrointestinal distress. Mechanistic links between these diverse symptoms and *SYNGAP1* variants remain obscure, therefore, our goal was to generate a zebrafish model in which this range of symptoms can be studied.

**Methods:**

We used CRISPR/Cas9 to introduce frameshift mutations in the *syngap1a* and *syngap1b* zebrafish duplicates (*syngap1ab*) and validated these stable models for Syngap1 loss-of-function. Because *SYNGAP1* is extensively spliced, we mapped splice variants to the two zebrafish *syngap1a* and *b* genes and identified mammalian-like isoforms. We then quantified locomotory behaviors in zebrafish *syngap1ab* larvae under three conditions that normally evoke different arousal states in wild-type larvae: aversive, high-arousal acoustic, medium-arousal dark, and low-arousal light stimuli.

**Results:**

We show that CRISPR/Cas9 indels in zebrafish *syngap1a* and *syngap1b* produced loss-of-function alleles at RNA and protein levels. Our analyses of zebrafish Syngap1 isoforms showed that, as in mammals, zebrafish Syngap1 N- and C-termini are extensively spliced. We identified a zebrafish *syngap1* α1-like variant that maps exclusively to the *syngap1b* gene. Quantifying locomotor behaviors showed that *syngap1ab* mutant larvae are hyperactive compared to wild-type but to differing degrees depending on the stimulus. Hyperactivity was most pronounced in low arousal settings, and hyperactivity was proportional to the number of mutant *syngap1* alleles.

**Limitations:**

*Syngap1* loss-of-function mutations produce relatively subtle phenotypes in zebrafish compared to mammals. For example, while mouse *Syngap1* homozygotes die at birth, zebrafish *syngap1ab−/−* survive to adulthood and are fertile, thus some aspects of symptoms in people with *SYNGAP1-*Related Disorder are not likely to be reflected in zebrafish.

**Conclusion:**

Our data support mutations in zebrafish *syngap1ab* as causal for hyperactivity associated with elevated arousal that is especially pronounced in low-arousal environments.

## Background

SYNGAP1-related disorder (SYNGAP1-RD), also known as SYNGAP1 syndrome, is caused by genetic variants in the *SYNGAP1* gene and is one of the most prevalent genetic forms of intellectual disability (ID) (Hamdan et al., 2010, 2011; Berryer et al., 2013; Satterstrom et al., 2020; Fu et al., 2022). Since the first patient report in 2009, there are now about ~1,400 known SYNGAP1 patients worldwide, though with advocacy and awareness, these numbers continue to rise (curesyngap1.org; May 2024). While ID and epilepsy are the most penetrant symptoms, some people with SYNGAP1 RD also present with Autism Spectrum Disorder (ASD; ~50%), gastrointestinal distress (~68%), developmental delay, hypersensitivity to sound and light, high pain thresholds (~72%), and challenging behaviors that include increased risk-taking, aggression, and self-injury (~73%) (Pinto et al., 2010; Carvill et al., 2013; Kilinc et al., 2018; Weldon et al., 2018; Jimenez-Gomez et al., 2019; Vlaskamp et al., 2019; Naveed et al., 2023; Thomas et al., 2024). The majority of SYNGAP1-RD-causing variants are *de novo*, occurring in the child but not in their parents (Hamdan et al., 2010, 2011; Berryer et al., 2013); SYNGAP1-RD is caused by haploinsufficiency, therefore, being heterozygous for a *SYNGAP1* variant can be sufficient to cause symptoms, with the median age of seizure onset being two years (Vlaskamp et al., 2019).

To better understand genotype/phenotype relationships in SYNGAP1-RD, we generated loss-of-function mutations in zebrafish *syngap1a* and *syngap1b* duplicates using CRISPR/cas9 (Varshney et al., 2016). We focused on translationally-relevant phenotypes in six-day-old larvae that correspond to early childhood in people. With accessible early development, optically transparent embryos, high fecundity, and established methods for genetic manipulation, zebrafish complement extant rodent models to understand the role of disease genes in development and behavior (Ijaz and Hoffman, 2016; Kozol et al., 2016; Thyme et al., 2019; Campbell et al., 2023; Weinschutz Mendes et al., 2023). While mammals have a single *SYNGAP1*, zebrafish *syngap1* is duplicated due to a whole genome duplication event 50–80 million years ago and retention of both *syngap1a* and *syngap1b* ohnologs (Glasauer and Neuhauss, 2014; Kozol et al., 2016). Two recent papers generated zebrafish *syngap1b* models that differ from the model we report here because they only target the “b” duplicate of the zebrafish *syngap1* ohnologs (Colon-Rodriguez et al., 2020; Griffin et al., 2021).

Mammalian *SYNGAP1* mRNAs are extensively spliced at N- and C-termini and the C-terminal isoforms α1, α2, β, and γ have been functionally characterized (Guo et al., 2009; McMahon et al., 2012; Araki et al., 2020; Kilinc et al., 2022). The α1 isoform is highly enriched at the post-synaptic density of glutamatergic synapses through a four amino-acid PDZ-interacting domain by which it interacts with the synaptic scaffolding protein PSD-95 (Chen et al., 1998; Kim et al., 1998; Komiyama et al., 2002). Here we annotate zebrafish mRNAs for how they might correspond to these mammalian isoforms and map zebrafish isoforms to *syngap1a* and *syngap1b* genes.

Individuals with SYNGAP1-RD show sensory hyperactivity, sometimes even seizures, in response to sensory stimuli such as eating, light, sound, touch, and/or pain (Vlaskamp et al., 2019). These symptoms are a major concern of the SYNGAP1 parents and caregivers because they can put these individual’s lives at risk (Vlaskamp and Scheffer, 2020; Lyons-Warren et al., 2022). Consistent with the human symptoms, rodent models also show sensory-induced hyperactivity as well as seizures that can be induced by loud sounds (Guo et al., 2009; Ozkan et al., 2014; Michaelson et al., 2018; Creson et al., 2019; Sullivan et al., 2020). To assess sensory-induced behaviors in *syngap1ab* zebrafish mutants, we used two standard sensorimotor assays: vibration to evoke the acoustic startle response (ASR) and transitions between light and dark to evoke the visual-motor response (VMR) (Emran et al., 2008; Gao et al., 2014; Dunn et al., 2016). Because changes to sensory habituation could contribute to sensory processing issues in SYNGAP1 patients (Tavassoli et al., 2014; Robertson and Baron-Cohen, 2017; Oldehinkel et al., 2019), we also conducted a short-term habituation assay to see how the *syngap1ab* zebrafish mutant larvae behave towards supra-threshold stimuli that are presented in rapid succession.

Like mammalian models, our zebrafish *syngap1ab* mutant models exhibit hyperactivity in both ASR and VMR assays. Hyperactivity was least pronounced in response to aversive acoustic stimuli and most pronounced during low-arousal light conditions. By analyzing the frequency distributions of movement distance and rest duration, we show that *syngap1ab* model hyperactivity in the light is due to higher-frequency, larger movements that resemble goal-directed behaviors associated with heightened states of arousal.

## Methods

### Fish maintenance and husbandry

All the zebrafish larvae and adults used in this study were reared at the University of Miami Zebrafish facility as per IACUC protocol #18–129. Water temperatures were maintained at 28°C and both adult and larval zebrafish were exposed to a circadian cycle of 14 h light/10 h dark. Water housing adult zebrafish was continuously monitored for pH and conductivity to maintain conditions within an optimal range (pH 7–8.1; conductivity 350–800 mOsm). Upon collection, embryos were rinsed briefly in reverse osmosis water and reared in 10 cm petri dishes containing ‘system water’ (taken from water housing adults). Dishes were cleaned daily to remove unfertilized eggs and prevent fungal growth that could limit oxygen and stunt early embryonic growth. Larvae used for behavioral assays were raised ~50 larvae per petri dish to minimize competition and developmental delays.

### CRISPR/Cas9 generation of *syngap1ab* mutant zebrafish

*Syngap1ab* mutants were generated using CRISPR/Cas9 genome editing technology (Varshney et al., 2016). One cell stage WT embryos were injected with small guide RNA (sgRNA; Integrated DNA Technologies-IDT Coralville IA) designed using CHOPCHOP online software (Montague et al., 2014) to target exon 4 of *syngap1a* gene (400 pg) and exon 5 of *syngap1b* gene (400 pg), along with Cas9 protein (PNA Bio Thousand Oaks CA; 100 pg). Embryos were either injected with *syngap1a* or *syngap1b* sgRNA using a foot-pedal-controlled Milli-Pulse Pressure Injector (MPPI-3 from Applied Scientific Instrumentation ASI Eugene OR). Resulting mosaic F0 larvae were reared to adulthood and crossed to wild-type animals to generate an F1 generation for Sanger sequencing to identify *syngap1a* and *syngap1b* mutant alleles with indels resulting from CRISPR editing (see below). Upon identification of the mutant *syngap1a* and *syngap1b* alleles, adults were in-crossed to obtain *syngap1ab* double mutants. Adult *syngap1a*b mutant fish, used to spawn larvae for experiments, span F2-F5 generations. To obtain *syngap1ab+/−* larvae, adult male *syngap1ab−/−* were outcrossed to WT (AB/TL, https://zfin.org/action/genotype/view/ZDB-GENO-031202-1) females. To obtain *syngap1ab−/−* larvae, *syngap1ab−/−* adults were in-crossed. For simplicity, zebrafish that are heterozygous in both *syngap1a* and *syngap1b* genes are denoted as *syngap1ab+/−*, and those homozygous in both *syngap1a* and *syngap1b* genes are denoted as *syngap1ab−/−.* All the wild-type (WT) larvae used for this study were AB/TL unless otherwise stated and are denoted as *syngap1ab+/+.*

### *Syngap1ab* alleles used in this study

The molecular identity of CRISPR alleles was determined by obtaining a small caudal fin sample from each anesthetized (200 mg/L of Tricaine (MS-222)) F1 adult fish. Genomic DNA was extracted by digesting each fin sample in 50 μL of 50 mM NaOH at 95°C for 1 h, the HotSHOT method (Samarut et al., 2016). To determine larval genotypes, each larva was anesthetized by either placing them on ice for 30 min or by using 200 mg/L Tricaine (MS-222). Upon complete anesthetization, larval genomic DNA (gDNA) samples were isolated using the HotSHOT method, as detailed above but using only 20 μL of 50 mM NaOH.

Gene-specific primers (Table 1) for both genes were designed using Primer3 software. Primer3 input sequences for each gene was selected based on their Cas9 target regions. For PCR, each reaction mixture contained 5 μL of 10x GOTaq Polymerase (Promega Madison WI), 0.5 μL from each 10 μM forward primer and reverse primer, 3 μL of nuclease-free water, and 1 μL of gDNA (from either larvae or adult-fin-clip digestions). Resulting PCR products were sent out for sequencing (Eurofins Genomics, LLC Louisville KY) and the results were read and analyzed using the ApE—A plasmid Editor v2.0.61 (Davis and Jorgensen, 2022) and SnapGene viewer software to determine the *syngap1a* and *syngap1b* mutant alleles (Supplementary Figure S1).

**Table 1:** *Syngap1* primers used for genotyping and qPCR.

Gene	Forward primer (5′–3′)	Reverse primer (5′–3′)
PCR primers used for genotyping		
*syngap1a*	GTGTTTTAGAGCACGCTCGTG	TCGAAATGGCTGTGTAGGGG
*syngap1b*	GTAGAAGACTGAAGGGGTCC	ACTTACCGCTCCTGGTCG
qPCR primers (From Kozol et al., 2016)		
*syngap1a*	CCTGAAGCTCATCGCACAC	GGGTCCACCTCACAGTTCTC
*syngap1b*	GACGACAGATCTCCATGCAC	GAGGAGTGGCGAGAGATGAA
*eef1a1*	CTGGAGGCCAGCTCAAACAT	ATCAAGAAGAGTAGTACCGCTAGC

### *Syngap1a* and *syngap1b* isoform identification

**Figure 1:**
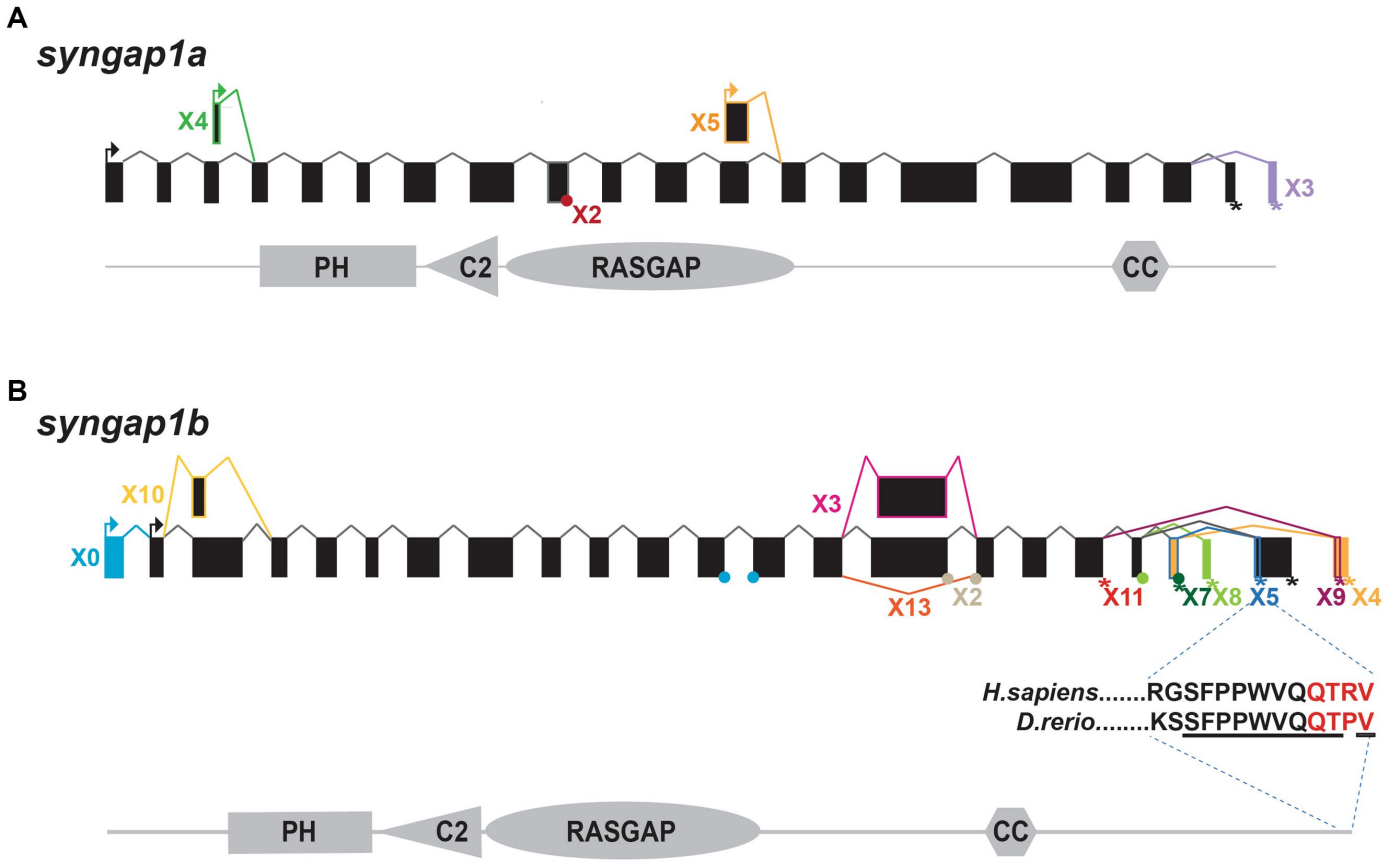
Zebrafish *syngap1a* and *syngap1b* isoforms. NCBI database searches revealed **(A)** five *syngap1a* (X1–X5) and **(B)** eleven *syngap1b* isoforms (X0, X2–5, X7–11, and X13) with evidence of expression. Transcriptional start sites are shown with arrows and stop codons are shown using an asterisk (*). Alternative splice sites are shown either using a dot (if the exon is missing <10 bp) or a box (if the difference is >10 bp). Isoform X*, box and connecting line colors denote exons and stop codons that are unique to the different isoforms. Black boxes and lines represent common exons/transcription start sites. The insert below the syngap1b X5 shows ten of eleven C-term amino acids (underlined) are identical to the human α1 isoform, with the PDZ-interacting domain shown in red.

To better characterize zebrafish *syngap1ab* splice variants, mRNA sequences (both published and predicted), were obtained from the NCBI protein database. To test for evidence of isoform expression, these sequences were searched against Expressed Sequence Tags (EST) and Transcriptome Shotgun Assembly (TSA) databases. Expressed isoforms were then BLASTed against the UCSC zebrafish genome browser to identify unique and common exons (Figure 1).

### qPCR

To check for nonsense-mediated mRNA decay in *syngap1ab* mutants, qPCR was used to quantify relative *syngap1ab* expression levels in *syngap1ab* mutant larvae compared to WT larvae, both groups at 7 days post-fertilization (dpf). To extract RNA, larvae were anesthetized by placing them on ice for 30 min before using TRIzol (Life Technologies, Carlsbad, CA) following manufacturer’s protocol. For each genotype (WT, *syngap1ab*+/−, and *syngap1ab*−/−), we conducted at least eight experimental replicates with 20 larvae pooled together per replicate. 1 μg of RNA was used as input for RT-PCR. To enhance syngap1 cDNA in each sample, *syngap1a* and *syngap1b* gene-specific primers were used with Eukaryotic translation elongation factor 1 like 1 *eef1a1l1* (ZFIN) as the internal control. cDNA was made using SuperScript III (Invitrogen™/ ThermoFisher Scientific) and incubating at 50°C for 1 h followed by 15 min at 70°C. qPCR was carried out using GoTaq qPCR Probe Kit (Promega) in a QuantStudio3 RT-PCR system (Applied Biosystems™, Waltham MA) following manufacturer’s protocol. Cycling conditions were as follows: Activation step of 95°C for 10 min, followed by PCR with 40 cycles of 95°C for 15 s and 60°C for 1 min, followed by a melt curve of 95°C for 15 s and 60°C for 1 min. Relative levels of gene expression were calculated using the ΔΔCt method. For this method cycle threshold Ct values for *syngap1a* and *syngap1b* genes were first normalized to Ct values of the internal control *eef1a1l1* by calculating ΔCt: Ct_syngap1a_-Ct_eef1a1l1_ and Ct_syngap1b_-Ct_eef1a1l1_. Fold-changes in *syngap1* gene expression were then compared in WT, *syngap1ab+/−*, and *syngap1ab−/−* larvae by calculating 2^-ΔΔCt^ with ΔΔCt:ΔCt_*syngap1ab* mutant_-ΔCt_WT_. Fold changes in *syngap1ab* mutants were calculated by dividing WT values. Group comparisons were made using 2-way ANOVA (gene and genotype) followed by Tukey’s multiple comparison test.

### Western blot analysis

Brain regions (mouse) or whole brains (zebrafish) were excised from C57BL6 mice or adult zebrafish. Tissues were lysed in 10 volumes (for zebrafish, each brain was considered ~15 mg) of lysis buffer (50 mM Tris pH 8.0, 100 mM NaCl, 1 mM EDTA, 1 mM EGTA, 1% TritonX-100, 0.2% SDS, 0.5% Sodium deoxycholate, with complete Protease inhibitor EDTA-free mix (Roche/MilliporeSIGMA Burlington MA)) using a Dounce homogenizer to obtain homogenized tissue samples. Each sample was then diluted by 1:10 using lysis buffer and ~ 20 μL from each sample was loaded into each gel lane. Based on these experimental settings, each sample, i.e., for both zebrafish and mouse tissue samples, contained about 29 μg of proteins per 20 μL. Samples were first probed with SYNGAP1 antibodies (Abcam ab3344 or NOVUS nbp2-27541) and were followed by probing with secondary antibodies (anti-rabbit IgG IRDye680; LICOR 926-68071 or anti-goat IgG IRDye680; LICOR 926-68074). Resulting signals were measured and imaged using the fluorescence-based Odyssey CLx Imaging System (LICORbio Lincoln NE).

### Analysis of *syngap1a* and *syngap1b*’s relative importance for survival

To test for the potential effects of *syngap1a* and *syngap1b* mutant alleles on larval survival, three batches of *syngap1ab+/−* in-crosses were genotyped at six dpf. Data were analyzed using Prism GraphPad software (v9.1) to determine whether observed allele representation differed significantly from predicted using a Chi-square test.

### Short-term habituation assay

To test whether the larvae show habituation in response to acoustic stimuli, a short-term habituation assay was used as described in Wolman et al. (2015). After 30 min of light adaptation, 6 dpf WT and *syngap1ab+/−* larvae were presented with 5 phases of acoustic stimulation using a DanioVision™ observation chamber (Noldus Leesburg VA). Phase 1 consisted of 10 tap stimuli (intensity level 3) delivered at a 20 s inter stimulus interval (ISI). Phase 2 consisted of 10 tap stimuli (intensity level 5) delivered at also at a 20 s ISI. Phase 3, the habituation test, consisted of 30 tap stimuli (intensity level 5) delivered at 1 s ISI. Phase 4 was a 3 min rest period. Lastly phase 5 consisted of 10 tap stimuli (intensity level 5), delivered at a 20 s ISI. Total distance moved by each larva per second was analyzed to measure short-term habituation. Data were analyzed using Prism GraphPad software (v9.1) to compare WT and *syngap1ab+/−* groups using a 2-way ANOVA (Phase and Genotype) followed by a Tukey’s multiple comparison test.

### Visual motor response (VMR) assay

*Syngap1ab* mutants and WT 6 dpf larvae larvae were placed in a 96-well plate containing system water. Prior to starting the experiment, larvae were dark adapted for 1 h and at 28°C in the Noldus DanioVision™ behavioral observation chamber. Ethovision® XT 11 software (Noldus) was used to program the delivery of stimuli and to analyze the results. Images were captured at 40 Hz. For the visual-motor response (VMR) assay, larvae were exposed to 5 min of 12% (high-light settings) lights-on stimulus followed by 5 min of lights-off stimulus. Larval movements were recorded for four consecutive light on/off cycles and their activity/movements were recorded for a total duration of 40 min. Larvae were then genotyped using the genotyping assays previously described and analyzed for the total distance moved/time. The resulting data were further analyzed using GraphPad Prism 9.1 software to compare WT, *syngap1ab+/−*, and *syngap1ab−/−* groups using Kruskal-Wallis ANOVA followed by Dunn’s multiple comparison tests.

### Displacement and dwell-time analyses

To test whether the observed hyperactivity is due to increased initiation of movements or increased distance moved, the displacement and dwell-time data from raw, exported data produced by Ethovision® XT 11 software were analyzed using MATLAB scripts^1^ to examine behavioral probability distributions. Resulting data were plotted using Prism GraphPad (V9.1) and WT and *syngap1ab* groups compared using a two-sample Kolmogorov–Smirnov test.

## Results

### Zebrafish *syngap1b* but not *syngap1a* encodes an isoform that is similar to the mammalian PDZ-interacting Syngap1α1

While mammals including humans have a single *SYNGAP1* gene that encodes a 1,343 amino acid, ~149 kDa protein (Komiyama et al., 2002), there are two *syngap1* ohnologs present in the zebrafish genome. The zebrafish *syngap1a* gene is found on chromosome 16 and encodes a 1,290 amino acid, ~146 kDa protein whereas the *syngap1b* gene is found on chromosome 19 and encodes a 1,507 amino acid, ~160 kDa protein. Like rodents and humans, both zebrafish *Syngap1a* and *Syngap1b* have four, highly conserved protein–protein interacting domains: pleckstrin homology (PH), C2, RasGAP, and coiled-coiled (CC).

We characterized zebrafish Syngap1 isoforms based on NCBI databases and comparisons to mammalian isoforms. In mammalian Syngap1, there are four well-characterized, alternatively-spliced Syngap1 isoforms, α1, α2, β, and γ, that vary at their C-termini (McMahon et al., 2012). To assess whether zebrafish *syngap1a* and *syngap1b* genes might encode similar isoforms, we curated all published and predicted *syngap1ab* isoforms. Similar to the mammalian isoforms, for both zebrafish *syngap1a* and *syngap1b* genes, exons encoding the four protein interaction domains occur in all isoforms with many alternative exons at both N- and C-termini. Based on expressed sequence databases, we were able to identify five *syngap1a* and eleven *syngap1b* isoforms (Figure 1). Of the human C-term isoforms, α1 is the most studied and is localized to the post-synapse by a four amino acid (QTRV) PDZ-interacting domain. Interestingly, we were able to find a zebrafish *syngap1b* C-term isoform X5 with a putative mammalian-like PDZ-interacting domain that had ten of eleven of the terminal amino acids identical to those of the human *SYNGAP1* α1 isoform (Figure 1B). We were unable to find mammalian-like isoforms (α2, β, and γ) due to the highly variable C-terminal ends in the zebrafish isoforms.

### CRISPR/Cas9-generated alleles produce loss-of-function SYNGAP1 models

**Figure 2:**
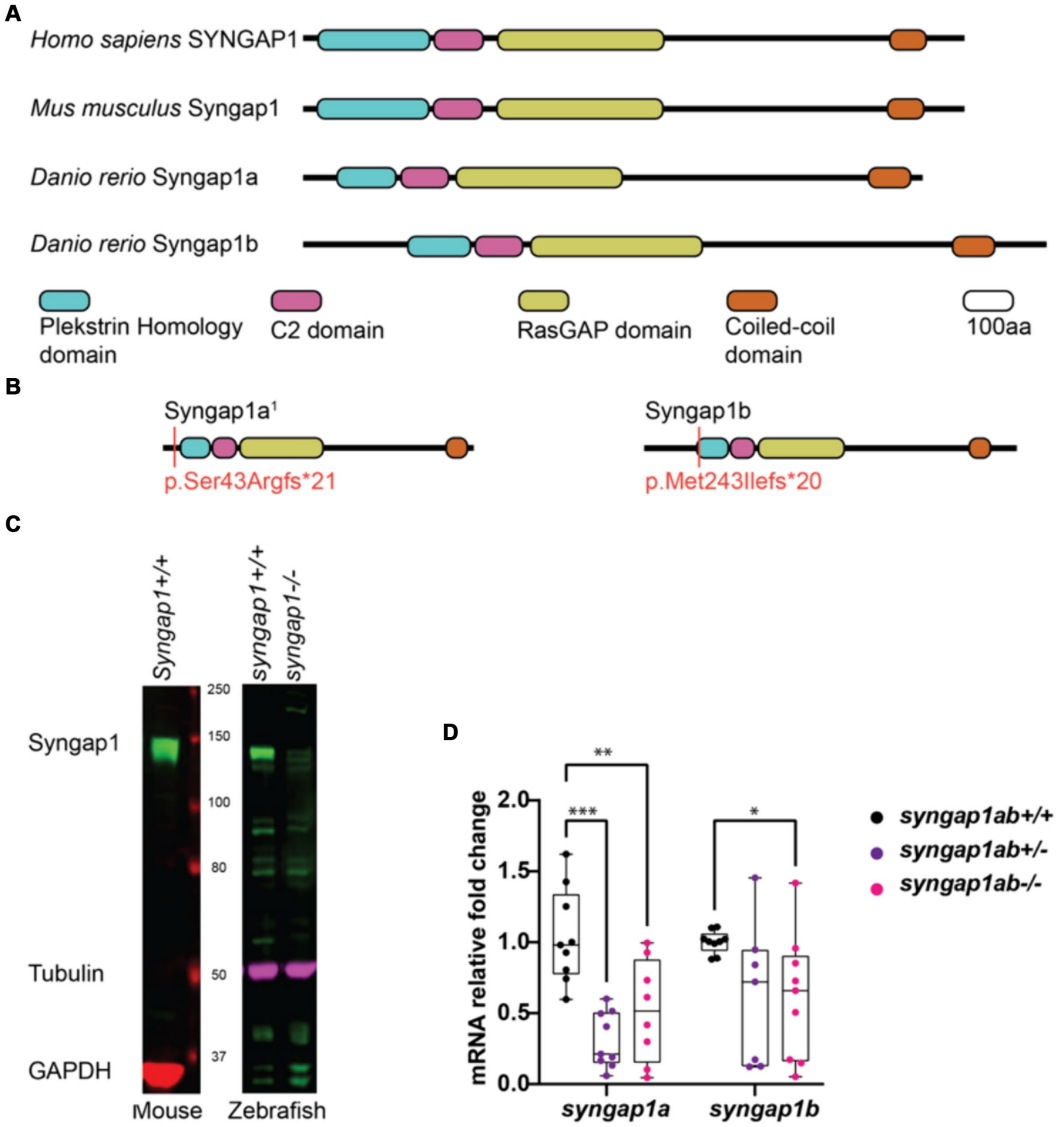
Zebrafish loss-of-function model for human SYNGAP1-RD. **(A)** Mammalian SYNGAP1 protein (*H. sapiens* and *M. musculus*) has four main protein interacting domains; pleckstrin homology (PH) domain, C2 domain, RasGAP domain, and coiled-coiled (CC) domain. Zebrafish (*D. rerio*) Syngap1 orthologs; Syngap1a and Syngap1b show domain conservation with that of mammals. **(B)** Syngap1ab protein diagrams show sites of CRISPR induced mutations. Resulting CRISPR mutants used for phenotypic analyses: *syngap1a*^1^ allele p.Ser43Argfs*21 and *syngap1b* allele p.Met149Ilefs*9. *syngap1a*^1^ had an amino acid change from a serine to an arginine at position 43 introducing a premature stop codon, 21 amino acids downstream. *Syngap1b* mutant allele had a change of methionine to an isoleucine at position 49 introducing a premature stop codon 9 amino acids downstream. **(C)** Western blots illustrate the expression of SYNGAP1 in whole brain lysates from adult mice and zebrafish. In wild-type zebrafish, the Syngap1 protein was detected at a similar molecular weight (~150 kDa) to that of the mouse SYNGAP1, using a rat anti-Syngap1 antibody. GAPDH and tubulin were used as the loading controls for mouse and zebrafish, respectively. **(D)** Mutant *syngap1ab* larvae showed reduced *syngap1a* and *syngap1b* mRNA expression levels at 7dpf. Group comparisons were made using 2-way ANOVA followed by Tukey’s multiple comparison test. *p* value asterisks represent: *p < 0.05, **p < 0.01; ***p < 0.001; ****p < 0.0001.

To better understand how pathogenic mutations in *syngap1* result in altered behaviors, we used CRISPR/Cas9 to generate zebrafish loss-of-function mutations. In humans, pathogenic variants span the *SYNGAP1* gene (Hamdan et al., 2011; Berryer et al., 2013; Vlaskamp et al., 2019; Gamache et al., 2020). To mutate a region of the gene that would affect the majority of isoforms, we targeted the earliest shared exons, exon 4 and exon 5 in *syngap1a* and *syngap1b* respectively, to generate loss-of-function alleles. CRISPR/Cas9 induces indels causing reading frame shifts and introducing premature stop codons. Upon sequence analyses of F1 adult crispants, we selected two mutant alleles for *syngap1a* (*syngap1a* + 7 nucleotide insertion and *syngap1a* − 22 nucleotide deletion) and one allele for *syngap1b* with a − 14 nucleotide deletion, all of which would be predicted to result in a severely truncated proteins that were less than 200aa (Figure 2B). To best recapitulate human SYNGAP1 variant haploinsufficiency, we used double-heterozygous larvae for *syngap1a* + 7 and *syngap1b* − 14, and to further assess complete loss-of-function, we used double-homozygous larvae (here onwards denoted as *syngap1ab*+/− and *syngap1ab*−/− respectively).

We tested for loss-of-function of *syngap1* at the level of protein and mRNA. Western blot analysis carried out using adult zebrafish brain lysates showed reduced Syngap1 protein levels in *syngap1ab*−/− brain tissues (Figure 2C). qPCR analysis of RNA harvested from 7-day-old larvae showed reduced RNA transcripts in both *syngap1ab+/−* (adjusted *p* values for *syngap1a* = 0.001, and *syngap1b* = 0.0495) and *syngap1ab−/−* (adjusted *p* values for *syngap1a* = 0.0699 and *syngap1b* = 0.0312) mutant larvae compared to WT larvae supporting non-sense mediated decay (Figure 2D). Taken together, these results show that our CRISPR/cas9 generated *Syngap1ab* zebrafish mutants show reduced mRNA and protein expression, supporting the use of these alleles as haploinsufficient models.

### Evidence for functional complementarity between *syngap1a* and *syngap1b* genes

To study potential interactions between Syngap1a and Syngap1b proteins, we analyzed larval survival from each of the nine genotypes resulting from *syngap1ab*+/− in-crosses. When *syngap1b* mutant alleles outnumbered *syngap1a* mutant alleles, larval survival was much lower than expected (Supplementary Figures S2A,B; *p* < 0.0001). By contrast, zebrafish in which there were more *syngap1a* than *syngap1b* mutant alleles were over-represented and zebrafish in which there were an equal number of *syngap1a* and *syngap1b* mutant alleles were as expected. These observations suggest that each ohnolog contributes distinct functional roles so that the *syngap1a* ohnolog, likely due to its inability to encode the Syngap1 α1 isoform, cannot make up for loss of *syngap1b* ohnolog.

### *Syngap1ab*+/− larvae show greater dynamic range, elevated response probability, and normal habituation in response to acoustic stimuli

**Figure 3:**
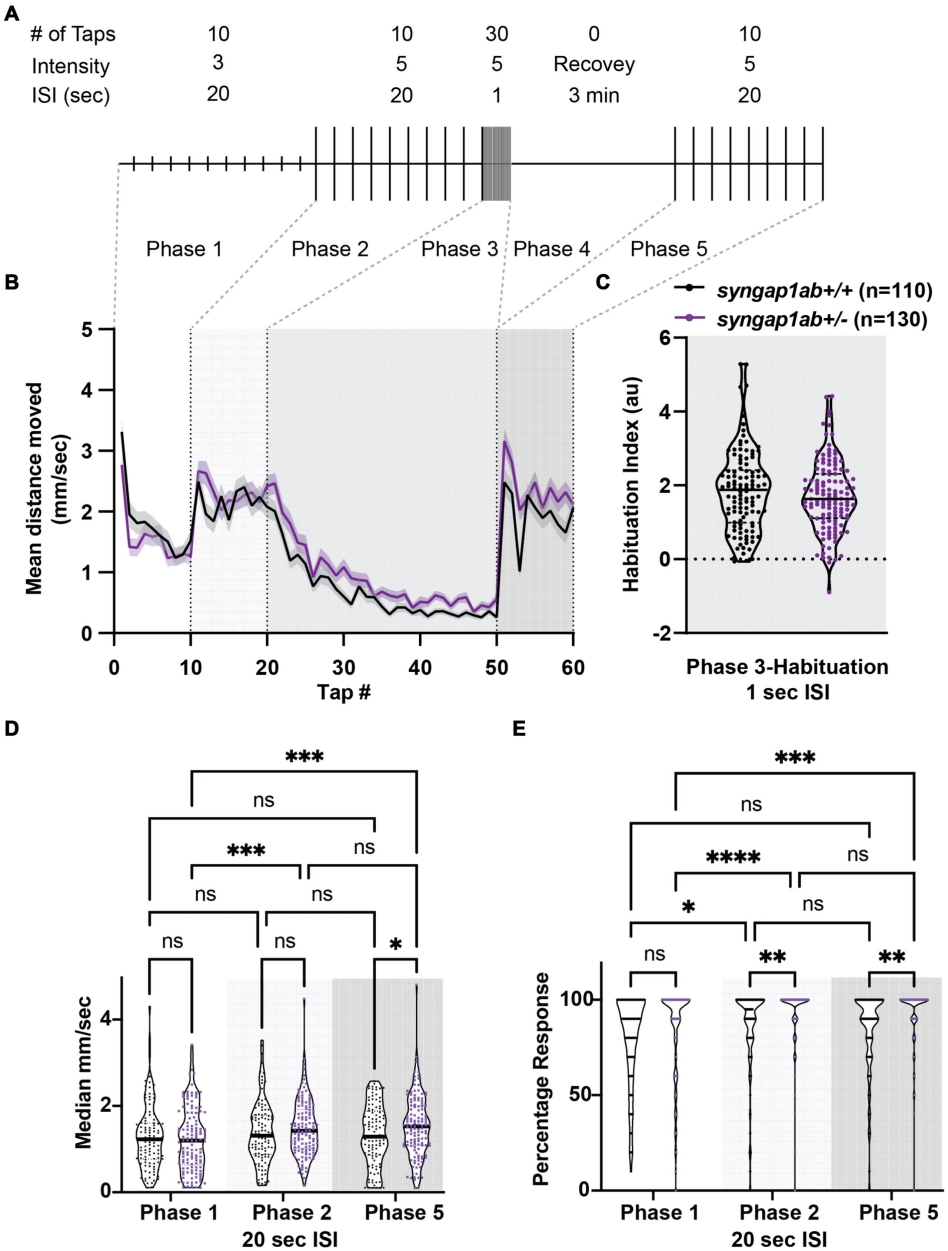
*Syngap1a*b larvae show short-term habituation to vibrational stimuli and more movement in response to stronger stimuli. **(A)** A schematic representation of the habituation assay (modified from Wolman et al., 2015) is shown. **(B)** Mean ± standard error movement velocity of WT and *syngap1a*b+/− larvae 1 s post-vibration. **(C)** Habituation indices were calculated per larva by subtracting the mean velocity post-habituation (taps 41–50) from the mean velocity pre-habituation (taps 11–20). **(D)** The median velocity mm/s moved per phase is shown for WT and *syngap1ab+/−* larvae. **(E)** Percentage response per phase is shown for WT and *syngap1ab+/−* larvae. A mixed effects model of genotype and phase was conducted and when *p* < 0.05 was followed by a Tukey’s multiple comparison test. *p < 0.05; **p < 0.01; ***p < 0.001; ****p < 0.0001.

Given that SYNGAP1 is known to be important for sensory processing, we wanted to test the dynamic range of *syngap1ab+/−* zebrafish responses to vibration stimuli of medium and high intensity as well as their ability to habituate to repeated, high-intensity, high-frequency stimuli. To assess these factors, we used an assay described in Wolman et al. (2015) in which 6 days-post-fertilization (dpf) larvae were exposed to different intensity vibrations with different inter-stimulus intervals (ISIs) during phases 1–5 of the assay (Figure 3A). We allowed larvae to acclimate to the Noldus chamber for 30 min before exposing them to vibrational stimuli. During phases 1 and 2, 10 taps of medium-intensity (level 3) and high-intensity (level 5) respectively were delivered with 20 s ISI. During phase 3, habituation was tested by delivering 30 high-intensity taps with 1 s ISI. This was followed by a 3 min rest period in phase 4. Finally, phase 5 was a repeat of phase 2 with 10 high-intensity taps delivered with 20 s ISI. Overall, *syngap1ab+/−* larvae (n = 130 from 3 independent crosses) responded very similarly to WT larvae (n = 110 from three independent crosses) (Figure 3B), with a normal degree of habituation to high frequency stimuli (Figure 3C).

Despite these similarities, there were subtle differences. *syngap1ab+/−* larvae showed consistently elevated responses to high-intensity stimuli during phases 2 and 5. To determine whether this was due to larger movements and/or an increased probability of response, we calculated median movement velocity per larva (Figure 3D) and their response probability (Figure 3E) during Phases 1, 2, and 5. For median movement velocity (calculated across taps in a given phase), a mixed effects model of phase and genotype indicated a significant effect of phase (*p* = 0.0018). The subsequent Tukey’s multiple comparison test showed that *syngap1+/−* larvae moved further in response to high-intensity vibrations in phases 2 and 5 than to medium-intensity vibrations in phase 1 (*p* = 0.0006 and 0.0007 respectively) while WT larvae moved similar distances during phases 1, 2, and 5. For probability of response, a mixed effects model indicated effects of both phase (*p* < 0.0001) and genotype (*p* = 0.0009). The subsequent Tukey’s multiple comparison test showed that *syngap1+/−* larvae had a higher response probability to stronger vibrational stimuli (*p* < 0.0001 Phase 1 vs. 2; *p* = 0.0002 Phase 1 vs. 5) and that this higher response probability to high-intensity stimuli was greater in *syngap1+/−* than WT larvae (*p* = 0.0049 Phase 2; *p* = 0.0014 Phase 5).

Therefore, in response to stronger stimuli*, syngap1*+/− are more likely to move and move faster, indicating a larger dynamic range of response in *syngap1+/−* larvae. This higher probability of response to the same stimulus is also consistent with higher levels of arousal in *syngap1+/−* larvae.

### *Syngap1ab* mutant hyperactivity is most pronounced in low-arousal settings

**Figure 4:**
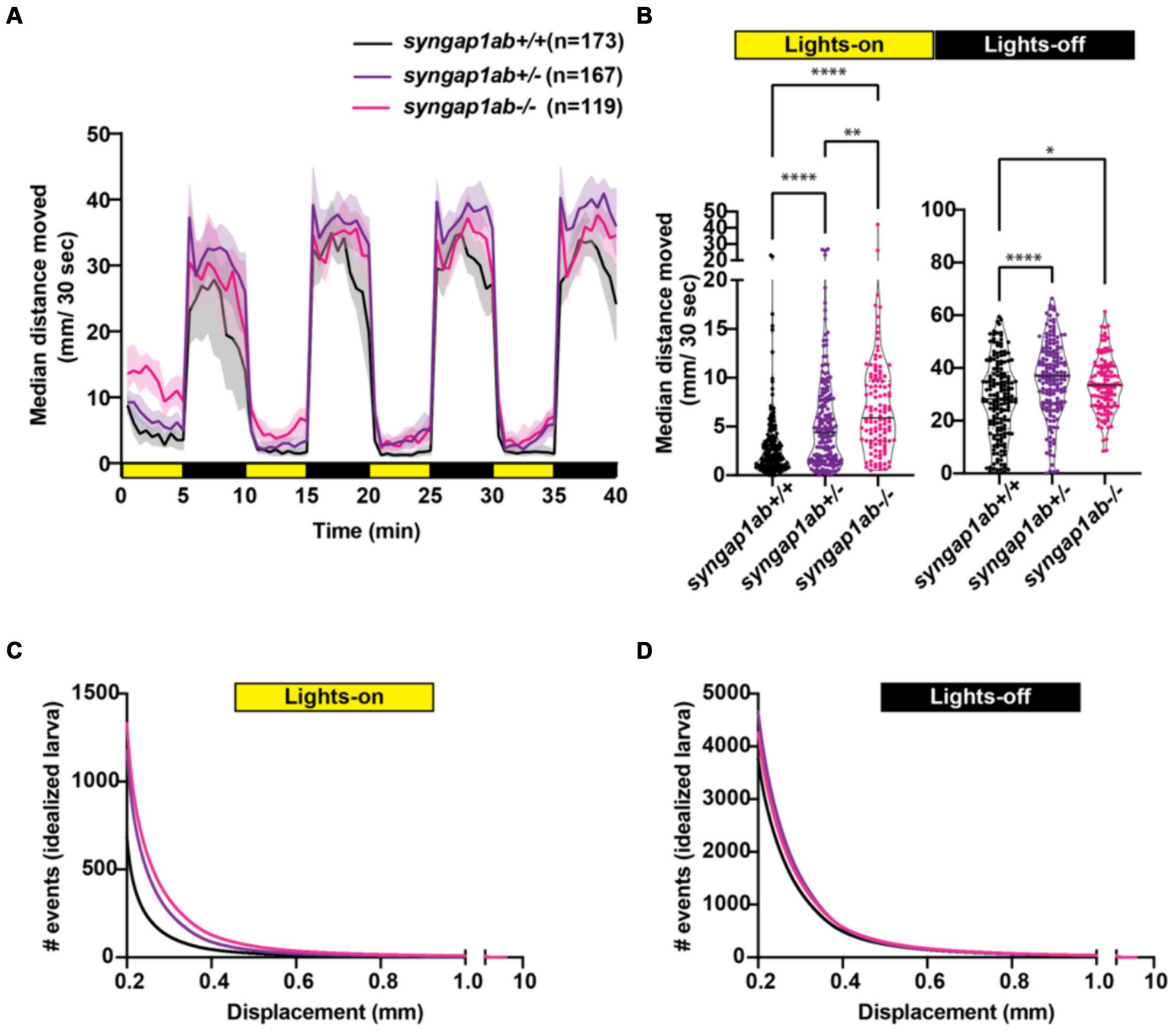
*Syngap1* model hyperactivity is most pronounced during light cycles due to a higher frequency of larger movements. **(A)** Median ± 95% confidence interval distance moved by each 6 dpf larva per 30 s, when exposed to 5 min of lights-on and 5 min of lights-off alternating cycles across five different independent trials (Supplementary Figure S3). **(B)** During lights-on cycles, *syngap1ab* mutants showed increased activity levels in a genotype dependent manner where *syngap1ab−/−* were more active than *syngap1ab+/−* which were more active than the WT larvae. During lights-off cycles, *syngap1ab* mutant larvae showed significantly increased activity compared to WT larvae but there were no significant differences in the activity levels between *syngap1ab−/−* and *syngap1ab+/−* larvae. Statistical analyses between genotypes were carried out using Kruskal-Wallis test followed by Dunn’s multiple comparison test. *p* value asterisks represent: *p < 0.05; **p < 0.01; ***p < 0.001; ****p < 0.0001. Displacement distribution of “idealized larva” during lights-on **(C)** and lights-off **(D)** conditions. Graphs were generated by pooling all displacement events during lights-on WT n = 119,135, *syngap1ab+/−* n = 196,594, and *syngap1ab−/−* n = 158,443 and during lights-off WT n = 652,368, *syngap1ab+/−* n = 777,525, and *syngap1ab−/−* n = 507,720 and then dividing these by the number of larvae: 173 WT, 167 *syngap1ab+/−,* and 119 *syngap1ab−/−*.

We next assessed visual-motor responses (VMR) (Burgess and Granato, 2007; Emran et al., 2008) in the *syngap1ab* models. For this assay, larval movements were recorded during four cycles of lights-on to lights-off transitions. Larval activity was measured as the total distance moved every 30 s. Larvae show robust increases in locomotor activity when presented with a sudden transition from light to darkness (Supplementary Figure S3; Figure 4A). Compared to WT larvae, both *syngap1ab+/−* and *syngap1ab−/−* larvae showed increased activity (*p* < 0.0001) during both lights-on and lights-off cycles (Figure 4B). During lights-off periods *syngap1ab+/−* showed the greatest movement but this trend was somewhat variable across different batches of larvae (Supplementary Figure S3B); during lights-on, hyperactivity was consistent across batches of larvae and was dependent on the number of mutant *syngap1* alleles with *syngap1ab−/−* showing the greatest movement (Figure 4; Supplementary Figure S3).

For the preceding assays, WT, *syngap1ab+/−*, and *syngap1ab−/−* larvae resulted from independent crosses. To rule out the influence of distinct parental genetic backgrounds on the observed behavior, we also examined VMR in larvae resulting from an in-cross between *syngap1ab+/−* adults (Supplementary Figures S2C,D). Consistent with the previous results, during both lights-on (Supplementary Figure S2C) and lights-off cycles (Supplementary Figure S2D), *syngap1ab−/−* had the highest activity levels among all the resulting genotypes.

### *Syngap1ab* hyperactivity in the light resembles WT behavior in the dark

The hyperactivity we observed in the *syngap1ab* mutant models could be due to either increased movement frequency, increased distance traveled per movement, or both. To distinguish among these possibilities, we analyzed the larval movement at a higher temporal resolution. Data were sampled every 25 ms, a much higher temporal resolution than the distance per 30 s shown in the preceding figures. Zebrafish movement bouts last ~250 ms and so 40 Hz resolution is sufficient to capture the majority of bouts with multiple timepoints. We focused on two parameters: the time interval between two consecutive movement bouts, denoted as “dwell time,” and the distance traveled per bout, denoted as “displacement.” These high-resolution activity data were organized and analyzed using custom-written MATLAB scripts to assess the probability of different behaviors, described by displacement and dwell time, during light and dark conditions.

Given highly stochastic individual larval movements, to assess overall patterns of both high and low frequency events across the distribution, we captured a large number of data points from over 100 individuals per genotype and then divided the total bouts by the number of individuals to generate and “idealized larva” for each genotype (Figures 4B,C). When bouts were pooled across individuals of a genotype, there were > 10^5^ bouts per genotype and light condition (Lights-On: WT n = 119,135, *syngap1ab+/−* n = 196,594, and *syngap1ab−/−* n = 158,443; Lights-Off: WT n = 652,368, *syngap1ab*+/− n = 777,525, and *syngap1ab*−/− n = 507,720) coming from 173 WT, 167 *syngap1ab*+/−, and 119 *syngap1ab*−/− individual larvae. This analysis shows that the differences between genotypes are much more pronounced in the light/low arousal settings, than in the dark/higher arousal setting.

**Figure 5:**
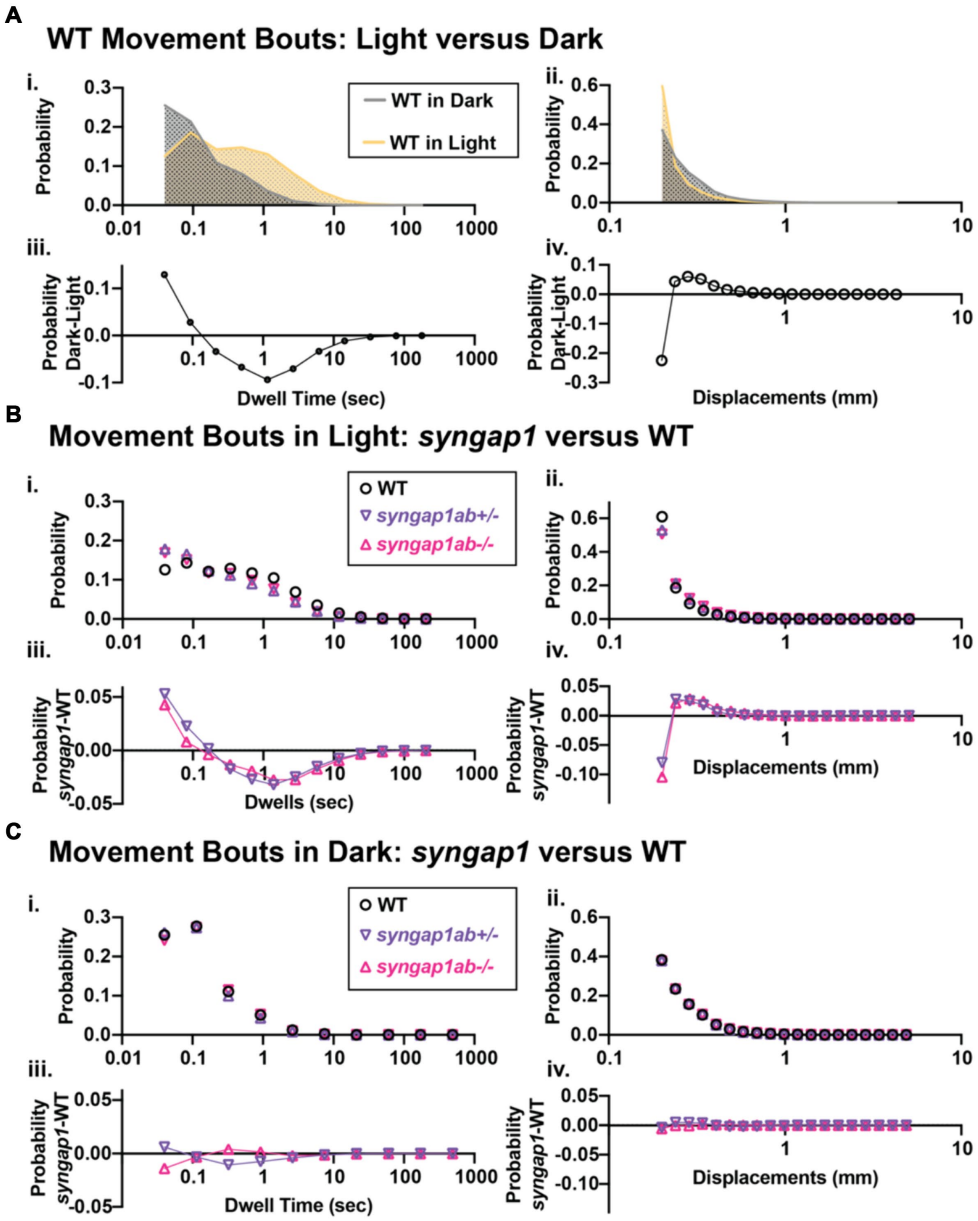
*Syngap1ab* mutants showed heightened arousal during lights-on cycles with more frequent and larger displacements. **(Ai,ii)** Probability distributions of dwell times **(Ai)** and displacements **(Aii)** are plotted for all 173 WT(*syngap1ab+/+*) larvae during lights-on (yellow) and lights-off (dark; checkered) cycles. Below **(Aiii,iv)** compare movements in dark and light by plotting probability in dark minus the probability in light. WT larvae moved farther more frequently in dark than in light. **(B,C)** Probability distributions of dwell time **(Bi, Ci)** and displacements **(Bii, Cii)** are plotted for all 173 WT, 167 *syngap1ab*+/− and 119 *syngap1ab*+/− mutant larvae. Below probability distribution plots **(Biii,iv, Ciii,iv)** compare movements in *syngap1ab+/−* (purple) and *syngap1ab−/−* (pink) to WT (black) by plotting probability in *syngap1ab* mutants minus the probability in WT. In dark, *syngpa1ab+/−* larvae moved more frequently than either WT or *syngap1ab−/−* larvae while all genotypes had similar displacement distributions. By contrast, in light, both *syngpa1ab+/−* and *syngap1ab−/−* larvae moved more frequently and farther than WT following a similar pattern that WT larvae showed during dark periods. *p* values were calculated using two-sample Kolmogorov–Smirnov test and for lights-on displacement: p(WT vs. *syngap1ab+/−*) = 0, p(WT vs. *syngap1ab−/−*) = 0, and p(*syngap1ab+/−* vs. *syngap1ab−/−*) = *p* < 10^−70^ and during lights-off displacement; p(WT vs. *syngap1ab+/−*) = 10^−26^, p(WT vs. *syngap1ab−/−*) = 1.5×10^−11^, and p(*syngap1ab+/−* vs. *syngap1ab−/−*) = *p* < 10^−45^, lights-on dwell time (WT vs. *syngap1ab+/−*) = 0, p(WT vs. *syngap1ab−/−*) = 0, and p(*syngap1ab+/−* vs. *syngap1ab−/−*) = *p* < 10^−31^ and lights-off dwell time; p(WT vs. *syngap1ab+/−*) = 10^−200^, p(WT vs. *syngap1ab−/−*) = 10^−50^, and p(*syngap1ab+/−* vs. *syngap1ab−/−*) = *p* < 10^−200^.

We next looked at probability distributions of dwell times (the time between movements) and distance traveled per movement (Figure 5). WT larvae in the dark had shorter dwell times and larger movements than WT in the light (Figures 5Ai,ii). These differences in WT light and dark behaviors are highlighted by plots below that show the relative probabilities in Dark versus Light for both dwell time and displacements (Figures 5Aiii,iv). Next, we compared *syngap1ab+/−* and WT in the light (Figure 5B) and in the dark (Figure 5C). In the dark, *syngap1ab+/−* and *syngap1ab−/−* had dwell time and displacement distributions that were very similar to WT larvae (Figures 5Ci–iv). By contrast, in the light, *syngap1ab−/−* and *syngap1ab+/−* mutants displayed both more frequent, and larger displacements compared to WT larvae (Figures 5Bi,ii). These differences between *syngap1+/−* and WT larvae in the light are highlighted by plots below that show the relative probabilities of *syngap1+/−* (purple) and *syngap1−/−* (pink) versus WT (Figures 5Biii,iv). These analyses showed that *syngap1*-WT (in light) resembles dark–light WT comparisons, indicating that *syngap1* mutants behavior in the light resembles that of WT behavior in the dark. Taken together, behavioral experiments show context-dependent hyperactivity that is most subtle during aversive, acoustic stimuli, is intermediate in the dark, and is most pronounced in normally low arousal well-lit environments.

## Discussion

In this study, we generated a zebrafish model of *SYNGAP1-RD* and characterized zebrafish *syngap1ab* splice-variants as they relate to mammalian *Syngap1* and zebrafish *syngap1a* and *syngap1b* duplicates. This provided background for the stable zebrafish mutant model of SYNGAP1-RD we generated using CRISPR/cas9 genome editing of both *syngap1a* and *synagp1b* duplicates; mutations were validated as loss-of-function alleles at both mRNA and protein levels. We provide evidence that *syngap1a* and *syngap1b* play complementary functional roles in zebrafish with higher larval mortality when *syngap1b* mutant alleles outnumber those from *syngap1a*. Focusing on balanced *syngap1a* and *syngap1b* mutant genotypes for more detailed analyses, we showed that, as in mammalian models and humans, the zebrafish *syngap1ab* models show context-dependent hyperactivity that is especially pronounced in low arousal settings.

Similar to mammalian Syngap1 isoforms (Gou et al., 2020; Yang et al., 2023), both Syngap1 zebrafish orthologs show extensive splicing at both N- and C-termini as well as splice-variants in the middle of the gene that only change a few amino acids. We provide evidence that *syngap1b* but not the *syngap1a* encodes an α1-like isoform, the most extensively studied of the mammalian Syngap1 isoforms (Chen et al., 1998; Kim et al., 1998; Walkup et al., 2016; Araki et al., 2020). In mammals, the α1 isoform has been shown to be enriched at the post-synaptic density of glutamatergic synapses by interacting with PSD-95 (Chen et al., 1998; Kim et al., 1998; Komiyama et al., 2002). In mice, loss of the α1 isoform alone is sufficient to produce cognitive deficits and seizures (Kilinc et al., 2022). The α1 isoform is also critical for long-term-potentiation-based forms of learning, toggling the strength of the synapse in an activity-dependent manner by competing with AMPA glutamate receptors for PSD95 binding sites (Araki et al., 2024). The unique expression of the Syngap1 α1 isoform by the *syngap1b* gene may help to explain higher mortality in zebrafish larvae with more *syngap1b* than *syngap1a* mutant alleles. We were not able to determine which of the zebrafish splice-variants corresponded to the other well-characterized mammalian Syngap1 α2, β, and γ isoforms (Kilinc et al., 2018; Araki et al., 2020; Gou et al., 2020), due to sequence divergence in the zebrafish consistent with differences that have been previously described in the zebrafish synapse proteome (Bayes et al., 2017).

Other zebrafish models of SYNGAP1-RD mutated only the *syngap1b* gene (Colon-Rodriguez et al., 2020; Griffin et al., 2021). Our differential survival results would predict that the phenotypes reported in *syngap1b* models may relate to a functional imbalance between *syngap1a* and *syngap1b*. Our more in-depth subsequent analyses, therefore, focused on larvae with balanced heterozygous mutations in both *syngap1a* and *syngap1b* in an effort to recapitulate mammalian haploinsufficiency.

In humans, altered sensory processing encompasses sensory hyperactivity/ hypoactivity and sensory seeking, and is a core symptom of an ASD diagnosis (Marco et al., 2011; Kirby et al., 2017; Robertson and Baron-Cohen, 2017; Damiano-Goodwin et al., 2018). In SYNGAP1-RD specifically, sensory-seeking behaviors include an affinity for contact with flowing water and/or perpetual motion (Wright et al., 2022). Syngap1 haploinsufficiency in both mice and humans alters sensory responses in a way that is context dependent, impacting simple sensory responses, entrainment, and habituation (Carreno-Munoz et al., 2022) and causing increased risk-taking behaviors (Kilinc et al., 2018; Weldon et al., 2018). Further studies on sleep in people with SYNGAP1-RD (Smith-Hicks et al., 2021) and mouse models, show disrupted sleep and seizures that are more common at night and often come in clusters that predict transitions between REM and non REM sleep (Sullivan et al., 2020). Taken together, this collection of symptoms is consistent with a heightened state of arousal and difficulties with behavioral state transitions in people with SYNGAP1-RD.

Similar to mammalian SYNGAP1-RD models, our *syngap1ab* zebrafish mutant models exhibit context-dependent hyperactivity. In high arousal contexts generated by strong, aversive acoustic stimuli, WT and *syngap1+/−* larvae produced similar highly-stereotyped, high-velocity escape responses, related to startle responses in mammals by their short latency from the stimulus and their dependence on reticulospinal neurons (Liu and Fetcho, 1999; Eaton et al., 2001; Korn and Faber, 2005). In contrast to WT, *syngap1ab+/−* larvae had a larger dynamic range of responses compared to WT. Larger displacements in response to aversive, acoustic stimuli have been described in zebrafish glucocorticoid receptor mutants that have chronically elevated glucocorticoids due to a lack of feedback inhibition (Griffiths et al., 2012). Unlike glucocorticoid receptor mutants however, our *syngap1ab+/−* model also moves faster and more frequently in the light. Therefore, *syngap1ab+/−* hyperactivity likely involves other arousal pathways.

In both mammals and zebrafish, arousal pathways including dopamine, QRFP, serotonin, and hypocretin/orexin among others have been linked to increased locomotion (Chiu and Prober, 2013; Lovett-Barron et al., 2017; Corradi and Filosa, 2021; Tan et al., 2022). Moreover, gain-of-function experiments in zebrafish have shown that overexpressing either hypocretin/orexin or CART (cocaine and amphetamine regulated transcript) is sufficient to increase the probability that zebrafish larvae will respond to acoustic stimuli (Prober et al., 2006; Woods et al., 2014); and overexpression of any one of hypocretin/orexin, calcitonin gene related peptide (cgrp), or cholecystokinin (cck) is sufficient to increase daytime movement frequencies (Woods et al., 2014). Thus, overactivation of select arousal pathways is consistent with *syngap1ab+/−* hyperactivity.

We found that *syngap1+/−* hyperactivity was greatest in the light, a setting characterized by low arousal, long dwell times and short movements in WT larvae. Both *syngap1ab+/−* and *syngap1ab−/−* larvae exhibited short dwell times and large movements, similar to WT movements when they are suddenly transitioned to the dark (Burgess and Granato, 2007; Emran et al., 2008; Kozol et al., 2021). Increased WT movements with sudden darkness have been shown to reflect a goal-directed, light-seeking behaviors also known as dark phototaxis (Horstick et al., 2017). Goal-directed increases in activity can also result from internal states, such as hunger, studied in 7-day-old zebrafish larvae that no longer have a yolk supply (Filosa et al., 2016; Wee et al., 2019). In this study, hyperactive *syngap1ab* mutants were 6-day-olds and therefore their hyperactivity was not likely to be driven by hunger. Hunger-induced hyperactivity is associated with reduced cortisol, increased activity in the serotonergic raphe neurons and increased risk-taking as larvae approach objects that could be either food items or predators (Filosa et al., 2016). Like hunger, flowing water can also evoke more frequent movements that are dependent upon serotonergic dorsal raphe serotonergic pathways (Yokogawa et al., 2012). It is possible that the hyperactivity that we observe in the *syngap1ab+/−* larvae in low arousal settings is a form of sensory-seeking behavior.

## Limitations

Zebrafish are less affected by Syngap1 loss-of-function than mammals. This difference suggests that phenotypes in zebrafish *syngap1ab* mutants might be less pronounced than symptoms in people with SYNGAP1-RD. Milder phenotypes in zebrafish than in mammals have been observed in several mutant models that affect synapses (Ono et al., 2001, 2002; Mongeon et al., 2008; Wang et al., 2008). One of the reasons for this is that at the time they are undergoing bursts of synaptogenesis, around birth in mice and around 3 dpf in zebrafish, they are vastly different sizes. Due to their relatively small size, zebrafish larvae do not need to breathe to supply their tissues with oxygen. For example, it is possible to generate a healthy, paralyzed, transparent zebrafish larva for *in vivo* imaging of physiological processes such as angiogenesis (Davis et al., 2021) because simple oxygen diffusion is sufficient to support oxidative processes. By contrast, in mice, the same mutations would prove lethal at birth when the pups have to breathe to provide oxygen to their tissues. These differences may help to explain why mutations in similar genes can be viable in the zebrafish model but more severe and/or lethal in the mammalian models.

## Conclusion

Studies in people with diverse genetic forms of neurodevelopmental conditions and in animal models show that, despite some shared aspects of etiology, including changes to neuro- and gliogenesis and altered excitatory/inhibitory balance (Hoffman et al., 2016; Willsey et al., 2021), detailed changes in neuroanatomy, behavioral profiles, and associated symptoms exhibit substantial differences by genotype supporting the existence of subtypes (Ellegood et al., 2015; Thyme et al., 2019; Zerbi et al., 2021; Zoodsma et al., 2022; Weinschutz Mendes et al., 2023). In the case of *SYNGAP1*, both human and animal model studies point to arousal pathways as playing an important role in context-dependent hyperactivity. These condition-specific phenotypes can serve as the basis for the development of precision therapies in animal models like zebrafish that are suited to high-throughput, high-content screening (Hoffman et al., 2016).

## Data availability statement

The datasets presented in this study can be found in online repositories. The names of the repository/repositories and accession number(s) can be found in the article/Supplementary material.

## Ethics statement

The animal study was approved by University of Miami IACUC protocol #18-129. The study was conducted in accordance with the local legislation and institutional requirements.

## Author contributions

SHS: Conceptualization, Data curation, Formal analysis, Investigation, Methodology, Resources, Supervision, Validation, Visualization, Writing – original draft, Writing – review & editing. SK: Data curation, Investigation, Project administration, Writing – original draft, Writing – review & editing. RK: Conceptualization, Methodology, Resources, Writing – original draft, Writing – review & editing. YA: Formal analysis, Methodology, Writing – original draft, Writing – review & editing. SS: Data curation, Formal analysis, Software, Visualization, Writing – original draft, Writing – review & editing, Funding acquisition. RH: Funding acquisition, Resources, Writing – original draft, Writing – review & editing. JD: Conceptualization, Data curation, Formal analysis, Funding acquisition, Investigation, Methodology, Project administration, Supervision, Validation, Visualization, Writing – original draft, Writing – review & editing.

## Footnotes

^1^ https://github.com/sheyums/Sureni_Sumathipala_syngap1.git
