# Supplementary material for: Context-dependent hyperactivity in syngap1a and syngap1b zebrafish models of SYNGAP1-related disorder
Source: Front Mol Neurosci. 2024 Jul 10;17:1401746. doi: 10.3389/fnmol.2024.1401746 (PMC11266194; doi:10.3389/fnmol.2024.1401746)
Supplement: Supplementary file 1 [file Data_Sheet_1.docx]

**
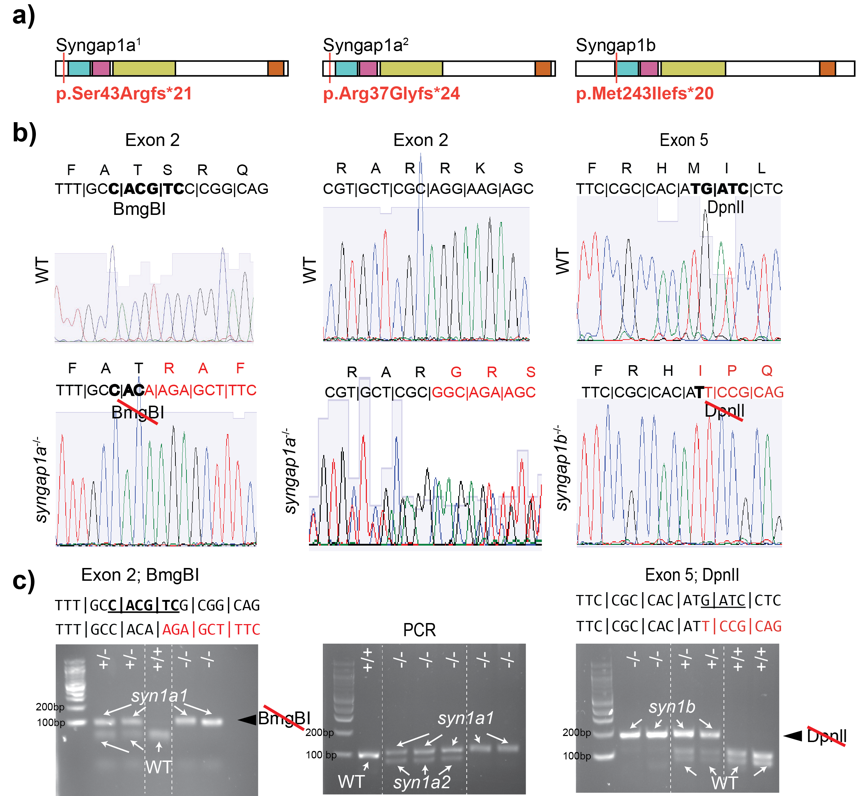
**

**Supplementary Figure 1 Genotyping zebrafish *syngap1a* and *syngap1b* mutant alleles.** **a)** Syngap1a and b protein diagrams are shown, one for each mutant allele, with red vertical lines indicating the location of each frame-shift mutation. Red type below the lines provides the details of the frameshift indicating the position of original amino acid, what it was changed to and the subsequent number of new amino acids before the stop codon truncation. Below each diagram are two electropherograms, the top showing the WT and the bottom showing the mutant sequences. Codons and corresponding amino acids are shown above each electropherogram. **b)** Genotyping gels are shown for each allele. For *syngap1a1*, a stretch of exon 2 that includes a restriction enzyme site for BmgB1 in the WT sequence (underlined) is destroyed in *syngap1a1*. *Syngap1a2* has a 22 base deletion that can be detected as a size shift directly from PCR amplification of Exon 2 surrounding the mutated site. For *syngap1b*, a stretch of exon 5 that includes a restriction site for DpnII in WT (underlined) is destroyed in the *syngap1b* mutant allele.

**
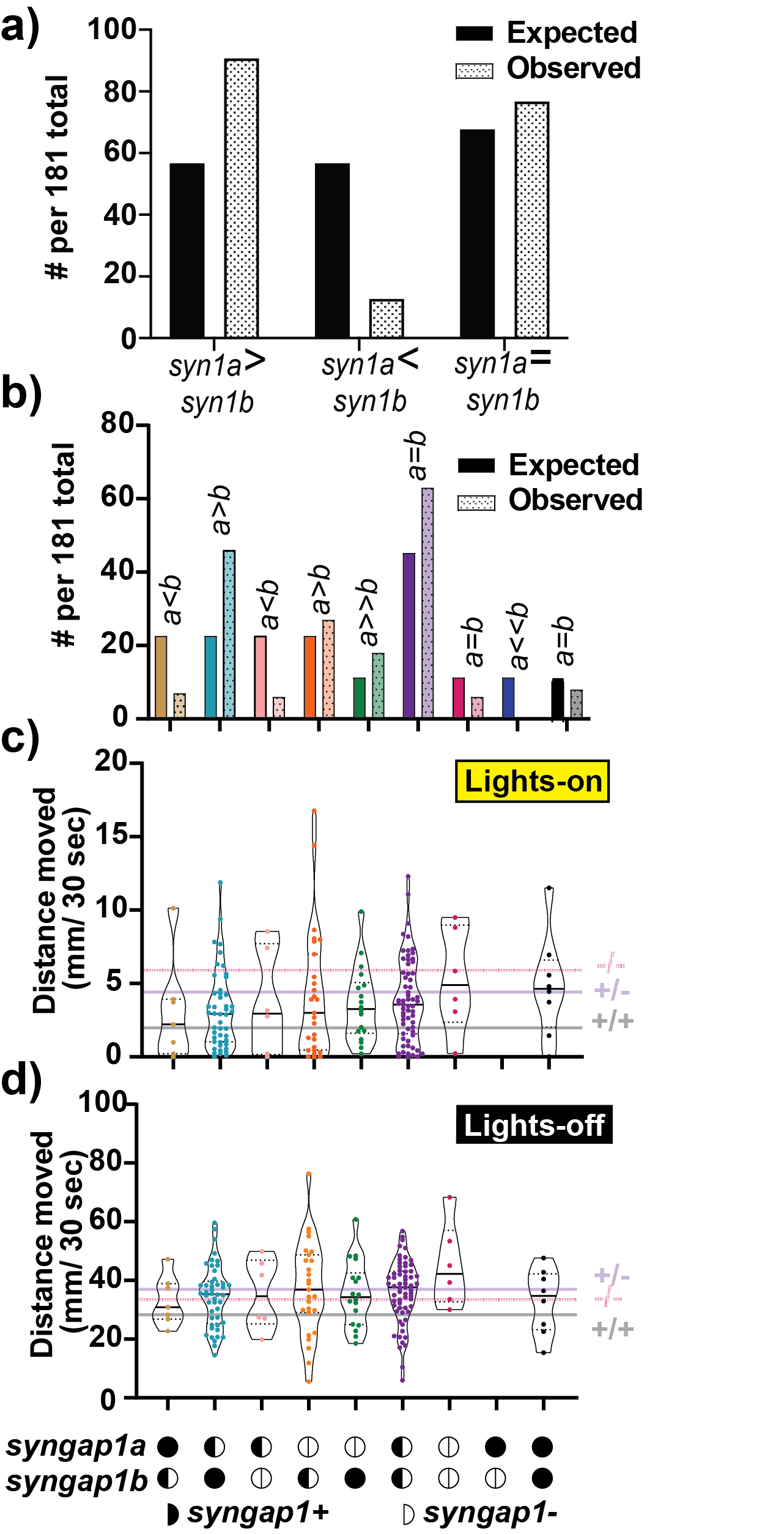
**

**Supplementary Figure 2 *syngap1b* is more important for larval survival than *syngap1a.* a & b)** Expected vs observed ratios of different *syngap1a* and *syngap1b* mutant allele combinations resulting from a *syngap1a*b+/- in-cross. A Chi-square test indicates that observed is different from expected ratios p<0.0001. **a & b)** Of note, allele combinations for which *syngap1b* mutant alleles outnumber those of *syngap1a* (a<b) are under-represented with the most extreme case being no surviving larvae with the *syngap1a+/+; syngap1b-/-* genotype. Median distance traveled in the light **c)** and in the dark **d)** are plotted against genotype for all allele combinations with the highest median distances seen in *syngap1a-/-;syngap1b-/-* larvae. Horizontal lines (gray WT, purple *syngap1ab+/-*, pink *syngap1ab-/-*) indicate medians from other analyses to contextualize values. Because of low representation of some genotypes, medians from the in-cross may not be representative.


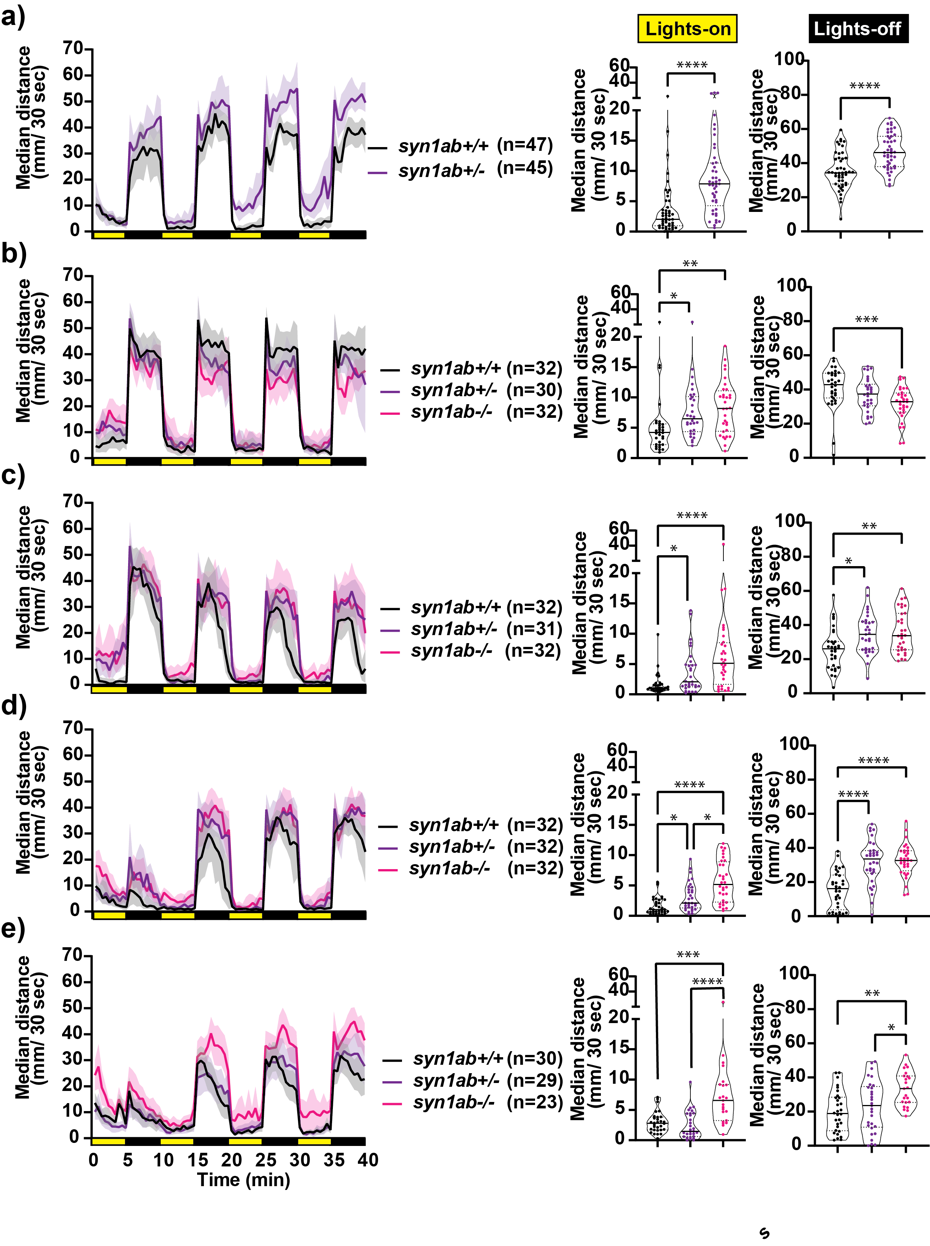


**Supplementary Figure 3 Visual Motor Response shows *syngap1* mutants are more consistently hyperactive in the light than in the dark. a-e)** Median + 95% confidence interval distance moved by each 6 dpf larva per 30 seconds, when exposed to 5 minutes of lights-on and 5 minutes of lights-off alternating cycles across five different independent trials sample size is indicated to the right of each VMR plot. To the right of that genotypes are compared in light and dark separately. In lights-on cycles, *syngap1ab* mutants showed increased activity levels in a genotype dependent manner where *syngap1ab-/-* were more active than *syngap1ab+/-* which were more active than the WT larvae. During lights-off cycles, syngap1ab mutant larvae often but not always showed significantly increased activity compared to WT larvae (see b). Statistical analyses between genotypes were carried out using Kruskal-Wallis test followed by Dunn’s multiple comparison test. P value asterisks represent; p<0.05 - *, p<0.01 - **, p<0.001 - ***, p<0.0001-******.**
